# Anterior Talo-Fibular Ligament Reconstruction With InternalBrace™ for Chronic Lateral Ankle Instability in Pediatric Patients

**DOI:** 10.7759/cureus.44979

**Published:** 2023-09-10

**Authors:** Mohit Sethi, Rajiv Limaye, Avinash Rai, Neil Limaye

**Affiliations:** 1 Orthopaedics and Trauma, North Tees and Hartlepool NHS Foundation Trust, Stockton, GBR

**Keywords:** mox-fq, visual analog scale (vas), chronic lateral ligament instability(clai), internal brace, anterior talofibular ligament

## Abstract

Introduction: Ankle injuries and instability in a pediatric age group are common problems and often underreported. The injuries can range from a relatively benign ankle sprain to pain-limiting ankle instability that can inhibit the child from participating in sporting activities. However, conservative management and physiotherapy are the mainstay of treatment; a small group of patients present with persistent instability and benefit from surgical intervention in lateral ligament reconstruction. Our study looked at pediatric patients who had instability following failed conservative management.

Methods: Retrospective analysis of 14 patients with Chronic lateral Ankle instability (CLAI) who underwent Modified Brostrom-Gould repair( MBG) with or without Internal brace augmentation between January 2015 and October 2020. Patients were evaluated for the visual analogue scale (VAS), Manchester-oxford foot questionnaire (MOxFQ), subjective satisfaction, and return to preinjury activity level.

Results: Pain score improved from 8 (average 5-9) to 1 (average 0-3) following surgery. Functional assessment was made by assessing the Manchester Oxford questionnaire pre-and postoperatively. MOxFQ scores improved from 64 (8 SD) to 7 (15 SD). Thirteen of fourteen patients returned to normal sporting activities at the final follow-up.

Conclusion: Modified Brostrom-Gould with InternalBrace™ augmentation is an excellent procedure for chronic lateral ligament injuries in the Paediatric population. It can be safely performed if we respect the anatomy and the physeal growth plate. It allows faster rehabilitation and return to preinjury activity level.

## Introduction

Acute ankle injury is one of the most common injuries seen in musculoskeletal practice, including both sports and non-sports-related injuries [1]. It forms a significant part of adolescent foot and ankle trauma. Ankle sprains comprise about 76% of injuries, followed by other musculoskeletal injuries. Lateral ankle sprains account for most ankle sprains, with 15-40% being sports injuries [2]. The remaining cases are either Salter and Harris type 1 injuries to the distal fibula or ATFL( Anterior talo-fibular ligament). Due to physis and lack of radiographic evidence, growth plate injuries should be excluded before considering ligament injury. With the advent of MRI, we pick up more ankle ligament injuries than ever before, and the myth that 'Children do not get sprains but fractures' is underreported. The Modified Brostrom-Gould (MBG) procedure with InternalBrace™ augmentation is well documented in the literature for the adult population but has not been published in the Paediatric population.

Acute lateral ligament ankle injuries are mostly managed conservatively with rest, ice, compression, and elevation, initially followed by ankle physiotherapy and rehabilitation with peroneal muscle strengthening and proprioceptive training. Conservative management of these injuries is effective in most of the cases. However, recurrent episodes of ankle sprain may lead to ligament attenuation or tear. In the long run, this can lead to chronic ankle instability [3]. Surgical management is the gold standard for those with failed conservative treatment for at least 12 months [3]. Brostrom described direct ligament repair that was anatomic. Brostrom technique gained popularity with improved biomechanical strength of the repair by 60% [4,5]. In addition, it enabled a full range of plantar and dorsiflexion and normal peroneal function [6]. Although MBG repair is widely accepted for chronic lateral ankle instability (CLAI), there are limitations to this technique. Several biomechanical studies [7,8] showed that the MBG procedure produces adequate clinical results but has significantly inferior strength compared to intact native ATFL. In his study, Kirk recommended protecting repaired ATFL from elongation during rehabilitation [8].

Moreover, the adequacy of repair, strength, and ability to resist forces is of doubtful significance, especially in patients with CLAI with attenuated native tissue and those with high-demand activities like athletes, high Body mass index, or hyperlaxity syndrome [9,10]. Furthermore, few studies showed significant postoperative improvement in outcome scores after the MBG procedure but had associated complications [11,12] and lower overall satisfaction scores [13]. Subsequently, there is increasingly a need to endorse the use of augmentation of repair to maintain stability and early rehabilitation and reduce the risk of complications [14]. Several techniques have been proposed to enhance the primary stability of repair or reconstruction, including free tendon grafts [10,15], suture anchors [13], or nonabsorbable tape augmentation [16-19]. An internal brace is a braided ultrahigh molecular weight polyethylene ligament used to augment the repair that acts as a secondary stabilizer [19]. The construct comprises fiber tape and two biocomposite Swivelock anchors. Several biomechanical studies show superior results with the use of Internal brace augmentation compared to MBG alone and MBG with suture tape augmentation [17] or native ATFL [18] and that the strength of the construct was like native ATFL [19]. Multiple clinical studies have recently shown good functional outcomes and early rehabilitation after MBG repair with the suture tape or Internal brace augmentation [16,20-25].

We present our small series of Fourteen children with chronic ATFL injury, which failed conservative treatment and underwent MBG with InternalBrace™ augmentation.

## Materials and methods

This study was conducted at the Department of Orthopaedics, University Hospital of North Tees, and Hartlepool Hospital, United Kingdom, between January 2015 and August 2020. There were 16 adolescent patients during this time frame who presented to A&E and were assessed for ankle injury as per standard protocol. Presenting symptoms and assessment protocol included 1) Reluctance to weight bear, 2) Tenderness and swelling over distal fibula, and 3) Radiographic orthogonal views to exclude ankle fracture. These patients were then referred to an orthopedic fracture clinic, where the on-call orthopedic team saw them. In the presence of tenderness over the distal fibula and clinical suspicion of a fracture, CT was requested to rule out undisplaced physeal injuries. Once fracture was excluded, these injuries were treated conservatively with rest, a walking boot, and a dedicated physiotherapy regime. Rehabilitation included ankle range of motion exercises, peroneal strengthening, and proprioception training. Patients were followed up at 8 weeks in a clinic for reassessment. In the presence of persistent pain and instability, patients were assessed clinically for laxity by checking the anterior drawer and calcaneal inversion. Weight-bearing/stress radiographs were also requested to confirm instability. An MRI was requested to rule out osteochondral lesions, ankle instability, and peroneal tendon pathology. Beighton's score was documented to rule out global instability. Where required, patients were also assessed for alignment problems by getting long leg views to rule out hip/knee pathologies. A detailed neurological examination was also carried out to rule out neurological problems and cavus foot. Once ligament injury was confirmed, patients were offered surgical treatment after 12 months of failed conservative management.

The inclusion criteria were skeletally immature patients with persistent symptoms at 12 months following injury and failed conservative management.

Surgical procedure

Anesthesia and Position

All surgeries were performed by the lead surgeon (RVL), a Consultant in Foot and ankle surgery. All procedures were performed under General anesthesia with the patient positioned supine on the table. A pneumatic thigh tourniquet and a sandbag were placed under the ipsilateral buttock.

Ankle Arthroscopy

Ankle arthroscopy was performed routinely using a 4.5 mm arthroscope and Ferkel ankle distractor. Standard anteromedial and anterolateral portals were used for joint access. Diagnostic ankle arthroscopy evaluated any underlying intraarticular pathology, including osteochondral lesions, syndesmotic injury, and synovial lesions.

Exposure

A longitudinal curvilinear incision was made over the distal fibula extending to the sinus tarsi. After subcutaneous fat and deep fascia dissection, the proximal border of the inferior extensor retinaculum was identified. Deeper dissection was then carried to the tip of the fibula and up to the neck of the Talus. In the case of attenuated ligament tissue, care was taken to protect the periosteum and inferior extensor retinaculum. Following this, peroneal tendons were inspected for tear nerve protection, and ATFL/CFL tear was confirmed.

Fibula Suture Anchors

Two 3.5 mm Twinfix anchors (Smith & Nephew, Andover, MA, USA) were used in the distal fibula to proceed with MBG repair. Care was taken to avoid physeal injury by taking appropriate X-rays when required. One suture anchor corresponded to ATFL, and the other to CFL fibular attachment. The suture anchor for ATFL was placed anterior to the Internal brace anchor.

MBG (Modified Brostrom-Gould) Repair

ATFL and CFL ligament imbrication was done using a horizontal mattress suture and reattached to a fibular footprint. If possible, periosteal flap imbrication was done, and the free edge of the Inferior extensor retinaculum was mobilized over ligaments and capsule and secured with sutures.

Internal Brace Application

For Internal brace augmentation, the first drill hole was made on the neck of the talus with a 3.4 mm drill at the insertion point of ATFL. After tapping, a 4.75 mm Biocomposite Swivelock anchor was inserted. The second drill hole was made at the footprint of ATFL on the distal fibula, followed by a 3.5 mm tap. The internal brace was then anchored in the distal fibula with a 3.5 mm Swivelock anchor. During the insertion, the ankle was kept in neutral dorsiflexion and slight eversion to get the optimum tension of the repair, taking care to allow 1-2 mm play in the internal brace to allow physiological movement of repaired ligaments. This was followed by MBG repair, as described above.

Postoperative Management and Rehabilitation

Intraoperative image intensifier X-rays were taken as a routine to ascertain the adequacy of the position of suture anchors and internal brace. Anterior drawer tests pre and post-repair were compared on the operating table. Postoperatively, patients were put into a back slab for two weeks and were kept non-weight-bearing for six weeks. At two weeks, sutures were removed, and the backslab was changed into a brace. All the patients were prescribed anti-coagulation according to their weight and age. Patients were assessed in the clinic at 6 weeks for stability, and weight bearing was commenced. A physiotherapy referral was made, and physio in the form of a range of motion exercises, everts or strengthening, and proprioception training was started. A strict rehabilitation protocol was followed after surgery, as shown in Table 1. The rehabilitation program was carried out under the supervision of physiotherapists with the aim of early rehabilitation and return to preinjury activity level. The contributing authors assessed patients at each follow-up, and data was entered into pre-set outcome sheets. PROMS were used to collect pre and postoperative data, and the VAS score was used to assess pain. Patient satisfaction rate was also documented. Patients complaining of persistent pain and instability were regarded as failures. It was clinically assessed with anterior drawer test and anterolateral ankle joint pain. Return to pre-injury activity was also assessed to look at rehabilitation time. Patients unable to return to pre-injury activity levels were also regarded as failures.

**Table 1:** Rehabilitation Protocol POP- Plaster of Paris; NWB- Non-weight bearing; PWB- Partial weight bearing; PF- Plantar flexion; DF- Dorsiflexion; ROM- Range of motion

Stage (weeks)	Brace	Weight Bearing	Rehabilitation Protocol	Goals
0-2	POP Back Slab	NWB	Prophylactic and general maintenance exercises (toes, knee, and hip ROM exercises)	Independent on crutches
					Advice regarding elevation	Prevent post-op complications
							02-Apr	Aircast boot	01/03/1952 NWB	Avoid PF > resting position i.e. plantigrade position i.e. plantigrade for 4 weeks	Reduce swelling
			01/04/1952 PWB	Prophylactic and general maintenance exercises (toes, ankle, knee and hip ROM exercises)	Pain control						
						Pain and edema control						Prevent scar adherence
Regular mobilisation of intermetatarsal and midtarsal joints (no subtalar or talocrural mobilization)	Start PWB at week 4 after surgery											
		Isometric exercises				Education on rehabilitaion program		
				Intrinsic muscle strengthening exercises Gradual progression to PWB by 4 weeks				
									04-Jun	Aircast boot	FWB	Gait re-education	Minimal swelling/ pain
		Gradual increase of ROM PF/DF				Full ROM PF/DF							
Resisted exercise for PF/DF	FWB in Aircast Boot										
		Proprioception Hydrotherapy									
06-Aug	Aircast boot	FWB		Promote normal gait	Normal gait pattern						
						Resisted exercise						No swelling and pain
				Closed chain exercises	Full muscle strength by 8 weeks							
						Proprioception			Abe to single stance>30 s
										Power walking on treadmill
				Increased CV work cross trainer/bike/rower pool						
					08-Dec	Aircast boot				FWB	Running initially on treadmill progress to road running	Proprioception as contralateral
				Plyometrics									Unrestricted confident function
														Agility work	Symptom-free

Table 2 depicts the demographic characteristics of patients. The average age was 15 years, and most of these injuries were sports-related/ twisting injuries.

**Table 2:** Summary of demographic characteristics of patients. RTA- Road traffic accident

Variables	Patients (N=14)
Age in years	15 (11-16)
Male/Female	8/6
Interval (months)	10 (12-27)
Etiology of injury	
Twisting Injury	9
Sports Injury	4
RTA	1
Post-operative follow-up in months	21 (14-29)

## Results

Between January 2015 and August 2020, we included 16 patients during this time frame. Two patients were lost to follow-up and were not included in the study. Patients were from 11-16 years of age, with the average age being 15 at the time of surgery. There were six female patients and 8 male patients who underwent surgical treatment. Surgery was done approximately 12-27 months following injury, an average 10 months. The average follow-up was 21 (14-29) months. All the patients underwent Modified Brostrom Gould repair with internal brace augmentation. Particular attention was paid to the physis, and two anchors were inserted distal to the growth plate in the fibula. One patient had hypermobility; one had an associated syndesmotic injury (Stabilized at the time of surgery), and one underwent an AMIC(Autologous matrix-induced chondrogenesis) procedure for osteochondral lesion.

Outcome measures

The hospital database was used to collect all the clinical information and stored as predesigned data sheets by the contributing authors. The clinical information included pre-operative clinical status, demographic data, and postoperative outcomes. Weight-bearing radiographs during follow-up appointments at two weeks, six weeks, three months, six months, 12 months, and 24 months were also retrieved and stored on the hospital database. Patient-reported outcome measures(PROMS) were used to assess clinical outcomes. The visual analogue scale (VAS) was used to assess pain, and the Manchester -Oxford foot questionnaire (MOXFQ) score was used to assess pre and postoperative outcomes. The patient's subjective satisfaction at the most recent/final follow-up was also documented in the notes. The time to return to preinjury activity level (PAL), including sports activity, was equally assessed. VAS evaluated the pain with a score of 0 = no pain and 10 = worst imaginable pain and assessed both pre and postoperative follow-up. The MOXFQ score is a self-administered 16-item patient-reported outcome measure (PROM) developed and validated in studies assessing outcomes following foot and ankle surgery [26]. The MOXFQ score measures three domains, namely walking/standing (7 items), pain (5 items), and social interactions (4 items), that have shown excellent psychometric properties in terms of reliability, validity, and responsiveness [27,28]. Each item has a response on a 5-point Likert scale ranging from 0 (no limitation) to 4 (maximum limitation). Raw scale scores are then converted to a metric from 0 to 100, where 100 denotes the most severe. Moreover, patients were examined clinically with an anterior drawer test and inversion stress test to assess lateral ligament stability.

We asked the patients to report pain and stability before and after surgery. After 6 months of conservative management, patients were asked to report pain on a Visual analogue scale. The same was recorded 6 weeks after surgery. Pain score improved from 8 ( average 5-9) to 1(average 0-3) following surgery. Functional assessment was made by assessing the Manchester Oxford questionnaire pre-and postoperatively. MOxFQ scores improved from 64 (8 SD) to 7 (15 SD). Thirteen of fourteen patients returned to normal sporting activities at the final follow-up. One patient still felt weak/unstable at a 17-month follow-up. Table 3 summarises the outcome of our study group.

**Table 3:** Summary of Outcome in patients with Chronic lateral ankle instability. VAS- Visual analogue scale; MOXFQ- Manchester Oxford Questionnaire; PAL- Pre-injury activity level

Variables	Pre-operative	Post-operative
VAS scale	8 (6-10)	1 (0-2.5)
MOXFQ score	64 (52-76)	7 (0-23)
Return to PAL	-	13/14 patients
Time to PAL (in weeks)	-	12 (8-16)

## Discussion

Lateral ligament reconstruction for persistent instability is one of the most commonly performed surgeries in Foot and Ankle practice. Surgical management with various techniques has been described in the literature, including non-anatomic reconstruction, anatomic repair, or reconstruction, and repair with augmentation. Kondarsen et al. [28], in their study with 7-year follow-ups, owed 80% of patients showed improved outcomes with conservative treatment followed by supervised rehabilitation. A systematic review by Song et al. [29] recommended surgical treatment after 3-6 months of non-surgical treatment with CLAI indication on physical examination or imaging. Kerkhoffs et al. [30], in their meta-analysis comparing surgical and conservative treatment, found significantly better outcomes in the surgical group, particularly in four domains, including chronic pain, subjective or functional instability, recurrence, and return to preinjury level of sports. Several surgical techniques are described in the literature for the management of CLAI. Historically, the surgical treatment began with Non-anatomic ligament reconstruction using local tendons described by Nilsonne [31], Watson-Jones [32], Evans [33], and Christmas-snook procedures [34]. However, non-anatomic reconstruction was associated with altered ankle and subtalar joint kinematics and subsequent subtalar arthritis, graft laxity, and poor postoperative recovery [35-37]. Brostrom, in 1966 [38], proposed anatomic ligament repair, which Gould further modified in 1980 [4]. MBG technique has been a gold standard surgical procedure for CLAI popularized by Hamilton et al. [6]. Anatomic repair helps to restore near-normal anatomic characteristics and joint biomechanics. These anatomic repairs or reconstructions can be undertaken as an open or arthroscopic technique.

Recently, there has been an increased trend toward using arthroscopic anatomic repair or reconstruction for CLAI [39-42]. However, early procedures depended on the non-anatomic imbrication of soft tissue with the potential to alter the hindfoot biomechanics [39]. Multiple studies, cadaveric [40] and clinical [41,42], have shown positive results with arthroscopic ligament repair, although there is a higher rate of complications. Guelfi et al. [43], in their systematic review comparing arthroscopic and open repair, found a higher complication rate than open technique. Thus, arthroscopic lateral ligament repair indication is still evolving. In the present study, Diagnostic Ankle arthroscopy followed by open MBG repair with or without Internal brace augmentation was performed. Arthroscopic examination of the ankle joint before open repair given the high incidence of concomitant intraarticular lesions in CLAI [44]. A study by Ferkel and Chams [2] identified intraarticular pathologies in 95% of cases. Similarly, Lee et al. [45], in a review of 28 ankles with CLAI, reported a frequency of 7-100% of associated intraarticular lesions. Although MBG repair is widely accepted for CLAI, the repair is significantly weaker than native ATFL.

Moreover, the procedure may fail to yield requisite stability with scarred and weakened ligament remnants with an increased risk of recurrence. Waldrop et al. [7] and Kirk et al. [8], in their biomechanical investigation, found that Brostrom repair provides only 50% of the strength of the native ATFL. Several studies [11,12] showed significant postoperative improvement but reported several complications, including ankle instability and ligament tear or lower overall satisfaction score [13]. Also, the adequacy of repair and its strength and ability to resist forces is of doubtful significance, especially in patients with CLAI with attenuated native tissue and those with high-demand activities like athletes, high Body mass index, or hyperlaxity syndrome, wherein there is a likelihood of excessive stress on the ankle and thus ligaments [9,10]. In addition, early mobilization elongation of the repaired ligaments may be associated with ankle laxity and instability. Kirk et al. [8] in his study recommended the need to protect repaired or reconstructed ATFL from elongation. As a result, it is vital to endorse the augmentation technique along with standard repair or reconstruction to maintain stability, early rehabilitation, and reduce risks of complications. In the present study, we used Internal brace suture tape augmentation with MBG repair.

The present study found good to excellent outcomes using internal brace augmentation, as summarized by significant improvement in mean postoperative MOXFQ score. In their biomechanical study, Schuh et al. [18] revealed superior performance for the angle at failure and failure torque for the internal brace group compared to Brostrom repair or suture anchor reconstruction methods. Similarly, in their cadaveric study, Willegger et al. [19] showed similar biomechanical stability of the Internal brace augmentation construct compared to native ATFL. Viens et al. [20]found that Internal brace augmentation is stronger and stiffer than native ATFL at time zero. Several clinical studies [16,20-25] have reported improved functional outcomes and earlier rehabilitation using an internal brace to augment MBG repair in CLAI. In their retrospective study, Batra et al. [21] showed excellent outcomes with IB augmentation in most patients with better ankle stability and ability to perform daily activities, including sports. Few studies [21-25] compared the outcomes of the Brostrom procedure with or without an internal brace and reported significantly better or similar [46] functional outcome scores in the Internal brace augmentation group. Mackay et al. [47], in their review of ligament augmentation with an internal brace, showed that IB supported the early mobilization of the repaired ligament with minimal surgical morbidity and highlighted the application of IB for augmentation of ATFL Brostrom and in anterior cruciate ligament repair in the knee.

Coetzee et al. [20] concluded that IB augmentation is a safe and effective procedure with favorable functional outcomes preventing recurrent instability and early return to sports with a mean of 12 weeks. Moreover, Yoo and Yang [22] and Ulku et al. [24] concluded that Internal brace augmentation has significant superiority in terms of early rehabilitation and return to sports. Our previous study on adult patients showed promising results with Internal brace application [48]. This encouraged us to take up this study in adolescent patients and assess the clinical outcome.

There are several limitations of this study. Firstly, the study is a retrospective analysis with inherent biases and limitations. However, all patients were managed by a single surgeon senior author (RVL), using the same protocol and methods, thereby decreasing the influence of confounding factors. Secondly, the study has a short-term duration of follow-up. Internal brace augmentation is a relatively new concept, and its use is still evolving and appreciated by surgeons. Thirdly, we did not include the patients who underwent concomitant procedures for associated injuries like syndesmosis; therefore, our conclusion may not apply to these patients. Fourthly, the number of patients under consideration is less, and we need a bigger subgroup of patients to present robust evidence in a pediatric population. Nonetheless, our study had several strengths. The results after internal brace use in the pediatric population haven't been reported.

## Conclusions

Lateral ankle instability is a fairly common problem in young and sporty individuals. Not much has been written or reported in this area. Treatment is understated, given the associated physeal injury and open growth plates. In reality, these groups of patients are the most active, and operative treatment should be considered wherever required. Proper patient selection and respect for the anatomy is the key when treating these subgroup of patients. Modified Bostrom-Gould with InternalBrace™ augmentation is an excellent procedure for chronic ankle injuries in the Paediatric population. It allows patients to be mobilized early and considers the hyperlaxity concurrently in this age group, especially in girls and athletic groups.

## References

[1] Guillo S, Bauer T, Lee JW (2013). Consensus in chronic ankle instability: aetiology, assessment, surgical indications and place for arthroscopy. Orthop Traumatol Surg Res.

[2] Ferkel RD, Chams RN (2007). Chronic lateral instability: arthroscopic findings and long-term results. Foot Ankle Int.

[3] Gerstner Garces JB (2012). Chronic ankle instability. Foot Ankle Clin.

[4] Brostrom ¨ L (1966). Sprained ankles. VI. Surgical treatment of “chronic” ligament ruptures. Acta Chir Scand.

[5] Gould N, Seligson D, Gassman J (1980). Early and late repair of lateral ligament of the ankle. Foot Ankle.

[6] Aydogan U, Glisson RR, Nunley JA (2006). Extensor retinaculum augmentation reinforces anterior talofibular ligament repair. Clin Orthop Relat Res.

[7] Hamilton WG, Thompson FM, Snow SW (1993). The modified Brostrom procedure for lateral ankle instability. Foot Ankle.

[8] Waldrop NE 3rd, Wijdicks CA, Jansson KS, LaPrade RF, Clanton TO (2012). Anatomic suture anchor versus the Broström technique for anterior talofibular ligament repair: a biomechanical comparison. Am J Sports Med.

[9] Kirk KL, Campbell JT, Guyton GP, Parks BG, Schon LC (2008). ATFL elongation after Brostrom procedure: a biomechanical investigation. Foot Ankle Int.

[10] Girard P, Anderson RB, Davis WH, Isear JA, Kiebzak GM (1999). Clinical evaluation of the modified Brostrom-Evans procedure to restore ankle stability. Foot Ankle Int.

[11] Schenck RC Jr, Coughlin MJ (2009). Lateral ankle instability and revision surgery alternatives in the athlete. Foot Ankle Clin.

[12] Petrera M, Dwyer T, Theodoropoulos JS, Ogilvie-Harris DJ (2014). Short- to medium-term outcomes after a modified Brostrom repair for lateral ankle instability with immediate postoperative weightbearing. Am J Sports Med.

[13] Hassan S, Thurston D, Sian T, Shah R, Aziz A, Kothari P (2018). Clinical outcomes of the modified Brostrom technique in the management of chronic ankle instability after early, intermediate, and delayed presentation. J Foot Ankle Surg.

[14] Messer TM, Cummins CA, Ahn J, Kelikian AS (2000). Outcome of the modified Broström procedure for chronic lateral ankle instability using suture anchors. Foot Ankle Int.

[15] Matsui K, Burgesson B, Takao M, Stone J, Guillo S, Glazebrook M (2016). Minimally invasive surgical treatment for chronic ankle instability: a systematic review. Knee Surg Sports Traumatol Arthrosc.

[16] Coughlin MJ, Matt V, Schenck RC Jr (2002). Augmented lateral ankle reconstruction using a free gracilis graft. Orthopedics.

[17] Cho BK, Park KJ, Kim SW, Lee HJ, Choi SM (2015). Minimal invasive suture tape augmentation for chronic ankle instability. Foot Ankle Int.

[18] Schuh R, Benca E, Willegger M, Hirtler L, Zandieh S, Holinka J (2015). Comparison of Brostrom technique, suture anchor repair, and tape augmentation for reconstruction of the anterior talofibular ligament. Knee Surg Sports Traumatol Arthrosc.

[19] Willegger M, Benca E, Hirtler L, Hradecky K, Holinka J, Windhager R, Schuh R (2016). Biomechanical stability of tape augmentation for anterior talofibular ligament (ATFL) repair compared to the native ATFL. Knee Surg Sports Traumatol Arthrosc.

[20] Viens NA, Wijdicks CA, Campbell KJ, Laprade RF, Clanton TO (2014). Anterior talofibular ligament ruptures, part 1: biomechanical comparison of augmented Broström repair techniques with the intact anterior talofibular ligament. Am J Sports Med.

[21] Coetzee JC, Ellington JK, Ronan JA, Stone RM (2018). Functional results of open Brostrom ankle ligament repair augmented with a suture tape. Foot Ankle Int.

[22] Batra AV, Nicholson D, Rao P, O’Sullivan J (2018). Clinical outcomes of the open modified brostrom procedure with internal brace augmentation for lateral ankle instability. Orthop Muscular Syst.

[23] Yoo JS, Yang EA (2016). Clinical results of an arthroscopic modified Brostrom operation with and without an internal brace. J Orthop Traumatol.

[24] Xu DL, Gan KF, Li HJ (2019). Modified brostrom ¨ repair with and without augmentation using suture tape for chronic lateral ankle instability. Orthop Surg.

[25] Ulku TK, Kocaoglu B, Tok O, Irgit K, Nalbantoglu U (2020). Arthroscopic suture-tape internal bracing is safe as arthroscopic modified Broström repair in the treatment of chronic ankle instability. Knee Surg Sports Traumatol Arthrosc.

[26] Cho BK, Park JK, Choi SM, SooHoo NF (2019). A randomized comparison between lateral ligaments augmentation using suture-tape and modified Broström repair in young female patients with chronic ankle instability. Foot Ankle Surg.

[27] Li H, Zhao Y, Chen W, Li H, Hua Y (202089). No differences in Clinical Outcomes of suture tape augmentation repair versus Brostrom Repair surgery for Chronic lateral ankle instability. Orthop J Sports Med.

[28] Morley D, Jenkinson C, Doll H, Lavis G, Sharp R, Cooke P, Dawson J (2013). he ManchesterOxford Foot Questionnaire(MOXFQ): development and validation of a summary index score. Bone Joint Res.

[29] Dawson J, Boller I, Doll H, Lavis G, Sharp R, Cooke P (201121). The MOXFQ patientreported questionnaire: assessment of data quality, reliability and validity in relation to foot and ankle surgery. Foot (Edinb).

[30] Dawson J, Boller I, Doll H, Lavis G, Sharp R, Cooke P, Jenkinson C (2012). Responsiveness of the Manchester-Oxford Foot Questionnaire (MOXFQ) compared with AOFAS, SF-36 and EQ5D assessments following foot or ankle surgery. J Bone Joint Surg Br.

[31] Konradsen L, Bech L, Ehrenbjerg M, Nickelsen T (2002). Seven years follow-up after ankle inversion trauma. Scand J Med Sci Sports.

[32] Song Y, Li H, Sun C, Zhang J, Gui J, Guo Q (20197). Clinical guidelines for the surgical management of chronic lateral ankle instability a consensus reached by systematic review of the available data. Orthop J Sports Med.

[33] Kerkhoffs GM, Handoll HH, de Bie R, Rowe BH, Struijs PA (2007). Surgical versus conservative treatment for acute injuries of the lateral ligament complex of the ankle in adults. Cochrane Database Syst Rev.

[34] Nilsonne H (1932). Making a new ligament in ankle sprain. J Bone Joint Surg Am.

[35] Watson-Jones R (19558213). Fractures and other bone and joint injuries vol. 2.

[36] Evans DL (1953). Recurrent instability of the ankle: a method of surgical treatment. Proc R Soc Med.

[37] Chrisman OD, Snook GA (1969). Reconstruction of lateral ligament tears of the ankle: an experimental study and clinical evaluation of seven patients treated by a new modification of the Elmslie procedure. J Bone Joint Surg Am.

[38] Hennrikus WL, Mapes RC, Lyons PM, Lapoint JM (1996). Outcomes of the Chrisman-Snook and modified-Broström procedures for chronic lateral ankle instability. a prospective, randomized comparison. Am J Sports Med.

[39] Karlsson J, Bergsten T, Lansinger O, Peterson L (1988). Lateral instability of the ankle treated by the Evans procedure. a long-term clinical and radiological follow-up. J Bone Joint Surg Br.

[40] Sugimoto K, Takakura Y, Akiyama K, Kamei S, Kitada C, Kumai T (1998). Long-term results of Watson-Jones tenodesis of the ankle. Clinical and radiographic findings after ten to eighteen years of follow-up. J Bone Joint Surg Am.

[41] Shoji H, Teramoto A, Sakakibara Y, Kamiya T, Watanabe K, Fujie H, Yamashita T (2019). Kinematics and laxity of the ankle joint in anatomic and nonanatomic anterior talofibular ligament repair: a biomechanical cadaveric study. Am J Sports Med.

[42] Drakos MC, Behrens SB, Paller D, Murphy C, DiGiovanni CW (2014). Biomechanical comparison of an open vs arthroscopic approach for lateral ankle instability. Foot Ankle Int.

[43] Nery C, Raduan F, Del Buono A, Asaumi ID, Cohen M, Maffulli N (2011). Arthroscopic-assisted Broström-Gould for chronic ankle instability: a long-term follow-up. Am J Sports Med.

[44] Vega J, Golano P, Pellegrino A, Rabat E, Pena F (2013). All-inside arthroscopic lateral collateral ligament repair for ankle instability with a knotless suture anchor technique. Foot Ankle Int.

[45] Guelfi M, Zamperetti M, Pantalone A, Usuelli FG, Salini V, Oliva XM (2018). Open and arthroscopic lateral ligament repair for treatment of chronic ankle instability: a systematic review. Foot Ankle Surg.

[46] Wasserman LR, Saltzman CL, Amendola A (200435). Minimally invasive ankle reconstruction: current scope and indications. Orthop Clin North Am.

[47] Mackay GM, Blyth MJ, Anthony I, Hopper GP, Ribbans MJ (2015). A review of ligament augmentation with the Internal Brace™: the surgical principle is described for the lateral ankle ligament and ACL repair in particular, and a comprehensive review of other surgical applications and techniques is presented. Surg Technol Int.

[48] Jain NP, Ayyaswamy B, Griffiths A, Alderton E, Kostusiak M, Limaye RV (2022). Is Internal brace augmentation a gold standard treatment compared to isolated Modified Brostrom Gould repair for chronic lateral ligament ankle instability? effect on functional outcome and return to preinjury activity: a retrospective analysis. Foot (Edinb).

